# Appendiceal Mucocele Diagnosed in Patients with Inflammatory Bowel Disease Using Endoscopic Ultrasound

**DOI:** 10.1155/2012/849892

**Published:** 2012-07-03

**Authors:** Uni Wong, Peter Darwin

**Affiliations:** Division of Gastroenterology and Hepatology, University of Maryland School of Medicine, Baltimore, MD 21201, USA

## Abstract

When a bulging appendiceal orifice is observed during surveillance colonoscopy, the possibility of appendiceal mucocele must be considered. Appendiceal mucocele is a rare group of lesions characterized by mucinous distension of the appendiceal lumen with the dangerous potential to rupture, resulting in the development of pseudomyxoma peritonei. Early recognition and diagnosis of appendiceal mucocele can prevent the dreaded complication of pseudomyxoma peritonei but it requires a high index of suspicion. Patients with inflammatory bowel disease are at increased risk for colorectal neoplasm but neoplasm of the appendix is infrequently reported. We report two of the first cases of appendiceal mucoceles diagnosed in patients with inflammatory bowel disease using endoscopic ultrasound.

## 1. Introduction 

When a bulging appendiceal orifice is observed during surveillance colonoscopy, the possibility of appendiceal mucocele (AM) must be considered. AM is a rare group of lesions characterized by mucinous distension of the appendiceal lumen with the dangerous potential to rupture, resulting in the development of pseudomyxoma peritonei (PMP). PMP is associated with significant morbidity and mortality with 10-year survival rate of less than 50% [1, 2]. Symptoms of AM are frequently absent or nonspecific, and the diagnosis is often made as an incidental finding during evaluation of unrelated complaints. Whether there is a causal relationship between inflammatory bowel disease (IBD) and AM remains unclear. Some authors have speculated that inflammation and blockage at appendiceal orifice may play a role in pathogenesis of AM [3, 4], while others have suggested that appendiceal adenoma is a neoplastic manifestation of IBD [5]. Patients with IBD often undergo colonoscopy for surveillance or diagnostic purposes, and endoscopic ultrasound (EUS) is a valuable imaging modality that can be used to evaluate suspicious lesions of the appendix. Early recognition and diagnosis of AM can prevent the dreaded complication of PMP. Patients with IBD are at increased risk for colorectal neoplasm, but neoplasm of the appendix is infrequently reported [6]. We report two of the first cases of appendiceal mucoceles diagnosed in patients with inflammatory bowel disease using endoscopic ultrasound.

## 2. Case 1

**Figure 1:**
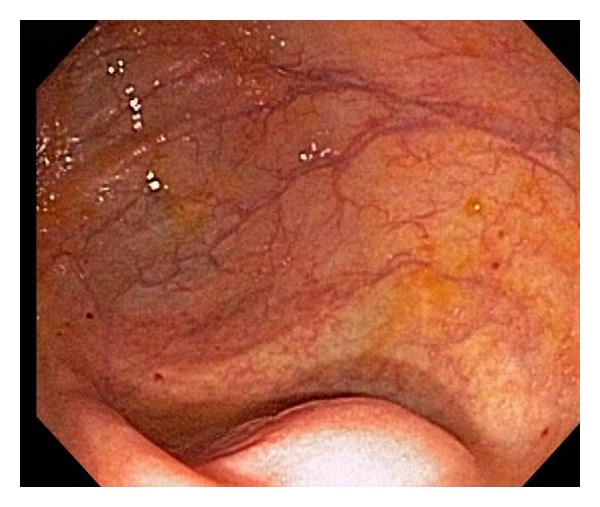
Protuberance at the appendiceal orifice seen at colonoscopy in Case 1.

**Figure 2:**
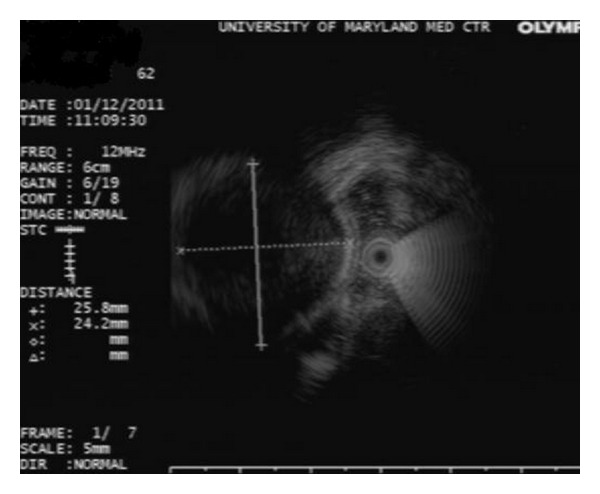
EUS image showing an anechoic structure within the appendix in Case 1.

 A 62-year-old female with ulcerative colitis in remission was found to have a 20 mm submucosal protuberance at the appendiceal orifice during surveillance colonoscopy (Figure 1). EUS (12 MHz TTS mini probe, Olympus America, Center Valley, PA) demonstrated a hypoechoic lesion with an anechoic heterogeneous center in the appendix (Figure 2), suggestive of mucocele. Patient underwent a successful laparoscopic appendectomy. Histology revealed mucinous cystadenoma of proximal appendix. 

## 3. Case 2

**Figure 3:**
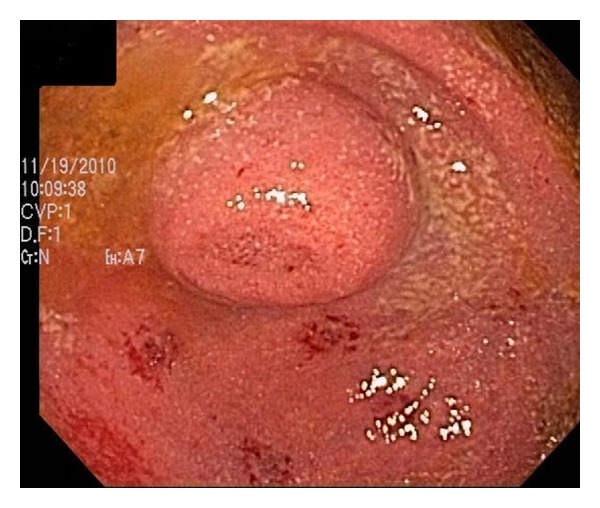
Protuberance at the appendiceal orifice seen at colonoscopy in Case 2.

**Figure 4:**
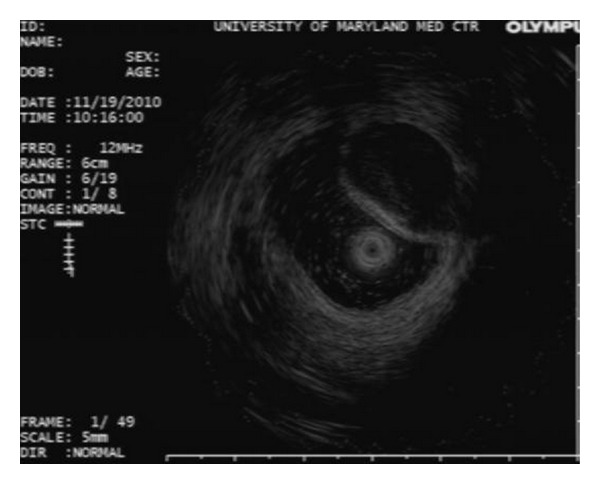
EUS image showing an anechoic structure within the appendix in Case 2.

**Figure 5:**
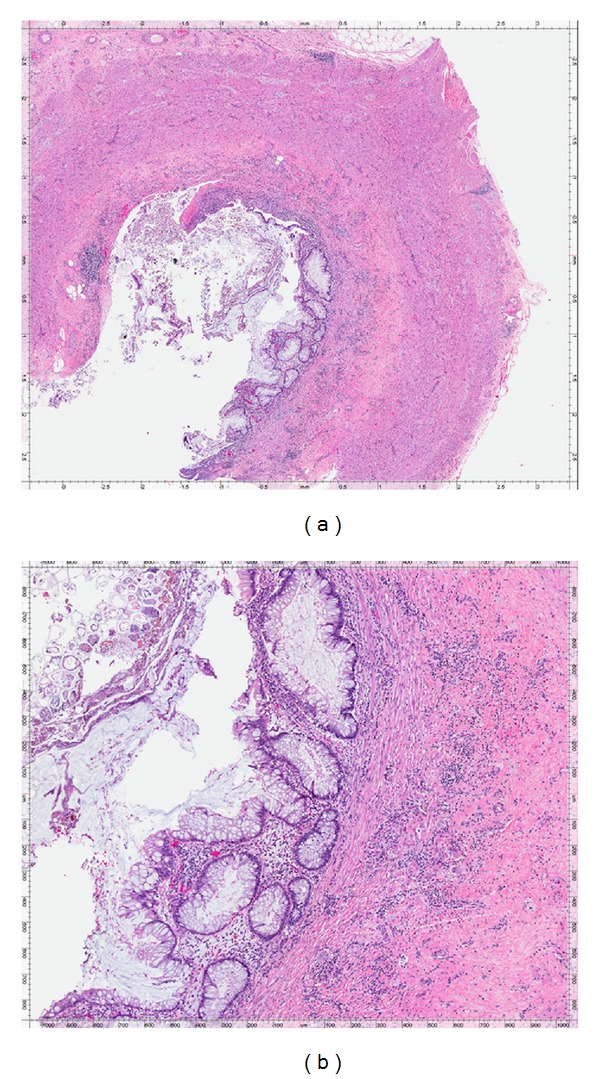
(a) Histological image of the appendiceal specimen in Case 2.  (b) Histological image at higher magnification demonstrating adenomatous changes at crypt bases with abundant mucin which is characteristic of appendiceal cystadenoma.

A 34-year-old female undergoing colonoscopy for evaluation of bloody diarrhea was found to have mild pan colitis (biopsy revealed ulcerative colitis) with an incidental finding of a bulging appendiceal orifice (Figure 3). EUS revealed an anechoic and homogeneous lesion measuring 20 mm in thickness with well-defined borders and lack of invasion to nearby structures (Figure 4). The mass was suspicious for appendiceal mucocele. Patient underwent appendectomy without any complications. Histological examination of the resected appendix revealed adenomatous changes with nuclear hyperchromasia and elongation, best seen at crypt bases with abundant mucin (Figures 5(a) and 5(b)). In addition, there is depletion of the normal underlying population of lymphocytes. This set of findings is consistent with the diagnosis of appendiceal mucinous cystadenoma.

## 4. Discussion

 The diagnosis of appendiceal mucocele must be considered when a distended appendiceal orifice is observed during colonoscopy because of its dreaded potential to cause pseudomyxoma peritonei. AM is a rare group of lesions found in only 0.3% of all appendectomies [7]. It is characterized by mucinous accumulation and distension of the appendiceal lumen. Four pathologic classes have been described: retention cyst, mucosal hyperplasia, cystadenoma, and cystadenocarcinoma, the latter two of which have the most potential to cause PMP if ruptured spontaneously or iatrogenically [8]. PMP is characterized by diffusing intra-abdominal gelatinous collections with mucinous implants on peritoneal surfaces and the omentum causing intestinal obstruction. The long-term survival in patients with PMP remains poor with reported 5- and 10- year survival rates of 50% and 10–30%, respectively [1]. Therefore, an accurate preoperative diagnosis of AM is crucial for optimal outcome.

 In a retrospective study consisted of 135 patients with AM, 55% were women [9]. Others, however, have reported a distinct male predominance of 3-4:1 [10, 11]. The patients in both cases described here are female. AM often presents as incidental findings without any clinical signs or symptoms, as demonstrated in the two cases here. Clinical manifestations of AM, when present, include palpable abdominal mass and abdominal pain at the right lower quadrant [3]. Other symptoms reported in other cases of AM include weight loss, nausea, vomiting, acute appendicitis, changes in bowel habits, and unexplained anemia [9]. Diagnosis of AM requires a high index of suspicion. While both cases of AM described here were diagnosed using EUS when a suspicious bulging appendiceal orifice was noted on colonoscopy, AM have been previously diagnosed incidentally on abdominal CT and abdominal ultrasound. AM can appear as a cystic well-encapsulated mass on abdominal CT and as a hypo-echoic or an anechoic cystic mass on abdominal ultrasound. Surgical resection is the current treatment of choice in the management of AM [9]. 

 The role IBD plays in AM remains unclear. Orta et al. observed a higher incidence rate of AM, particularly cystadenoma, in patients with IBD with concurrent colorectal neoplasms, speculating that appendiceal mucinous cystadenoma may be a neoplastic manifestation of colorectal cancers [3]. Others have suggested that obstruction of the appendiceal orifice might play a role in the development of AM, whether the blockage is due to inflammation in setting of IBD or an associated colorectal neoplasm [3, 4]. AM has also been reported in patients with endometriosis or carcinoid tumor-associated occlusion of lumen [8]. Neither of the cases described here had concurrent any colorectal lesions noted on colonoscopies. Patient in Case 2 had some inflammation around the appendiceal orifice along with pan colitis but patient in Case 1 was in remission of ulcerative colitis.

 EUS is a useful imaging modality to distinguish intramural from extracolonic lesions. It can also identify the echogenicity, architecture, and wall layer of origin of the lesion [12]. Optimal therapy of AMs requires an accurate preoperative diagnosis and careful resection in order to prevent the dreaded complication of pseudomyxoma peritonei. As illustrated in these two cases, AMs often present without preceding symptoms. Therefore, a high index of suspicion for AMs is important in patients with an abnormal appearing appendix and underlying IBD. Patients with IBD undergo surveillance endoscopy routinely, and EUS appears to be a useful imaging modality for evaluating subepithelial lesions in this setting.
